# Drug-eluting stents for coronary artery disease in the perspective of bibliometric analysis

**DOI:** 10.3389/fcvm.2024.1288659

**Published:** 2024-02-19

**Authors:** Ying Zeng, Jiawei Xu, Yuxuan Deng, Xiaoxing Li, Wen Chen, Yu Tang

**Affiliations:** ^1^ Jiangxi Medical College, Nanchang University, Jiangxi Provincial People's Hospital, The First Affiliated Hospital of Nanchang Medical College, Nanchang, China; ^2^ Department of Endocrinology and Metabolism, The First Affiliated Hospital, Jiangxi Medical College, Nanchang University, Nanchang, China; ^3^ The First Affiliated Hospital, Jiangxi Medical College, Nanchang University, Nanchang, China; ^4^ Jiangxi Cancer Hospital, Nanchang, China; ^5^ Jiangxi Provincial People's Hospital, The First Affiliated Hospital of Nanchang Medical College, Nanchang, China

**Keywords:** drug-eluting stents, coronary artery disease, bibliometric analysis, percutaneous coronary intervention, outcome, dual antiplatelet therapy

## Abstract

Drug-eluting stents (DES) play a crucial role in treating coronary artery disease (CAD) by preventing restenosis. These stents are coated with drug carriers that release antiproliferative drugs within the vessel. Over the past two decades, DES have been employed in clinical practice using various materials, polymers, and drug types. Despite optimizations in their design and materials to enhance biocompatibility and antithrombotic properties, evaluating their long-term efficacy and safety necessitates improved clinical follow-up and monitoring. To delineate future research directions, this study employs a bibliometric analysis approach. We comprehensively surveyed two decades' worth of literature on DES for CAD using the Web of Science Core Collection (WOSCC). Out of 5,778 articles, we meticulously screened them based on predefined inclusion and exclusion criteria. Subsequently, we conducted an in-depth analysis encompassing annual publication trends, authorship affiliations, journal affiliations, keywords, and more. Employing tools such as Excel 2021, CiteSpace 6.2R3, VOSviewer 1.6.19, and Pajek 5.17, we harnessed bibliometric methods to derive insights from this corpus. Analysis of annual publication data indicates a recent stabilisation or even a downward trend in research output in this area. The United States emerged as the leading contributor, with Columbia University and CRF at the forefront in both publication output and citation impact. The most cited document pertained to standardized definitions for clinical endpoints in coronary stent trials. Our author analysis identifies Patrick W. Serruys as the most prolific contributor, underscoring a dynamic exchange of knowledge within the field.Moreover, the dual chart overlay illustrates a close interrelation between journals in the “Medicine,” “Medical,” and “Clinical” domains and those in “Health,” “Nursing,” and “Medicine.” Frequently recurring keywords in this research landscape include DES coronary artery disease, percutaneous coronary intervention, implantation, and restenosis. This study presents a comprehensive panorama encompassing countries, research institutions, journals, keyword distributions, and contributions within the realm of DES therapy for CAD. By highlighting keywords exhibiting recent surges in frequency, we elucidate current research hotspots and frontiers, thereby furnishing novel insights to guide future researchers in this evolving field.

## Introduction

CAD, a cardiovascular ailment, exerts a profound global health impact. This malady stands as a principal contributor to mortality in both developed and developing nations (1–3). CAD's pathogenesis chiefly revolves around atherosclerosis development or coronary artery obstruction—a pathological sequence engendering constrained coronary blood flow. This impairment subsequently jeopardizes cardiac blood supply, potentially instigating manifestations like myocardial ischemia and angina pectoris. At its gravest, this process could culminate in myocardial infarction (4–7). The intricate etiology of CAD materializes as an intricate outcome stemming from multifaceted interactions. This intricate interplay emerges from a fusion of elements spanning lifestyle components (diet, exercise, smoking, alcohol consumption, etc.), environmental triggers (air pollution, psychological stress, etc.), and genetic predispositions (8–11). Percutaneous coronary intervention (PCI) stands as a widely employed method for treating coronary artery disease. This procedure involves the implantation of a stent within a coronary artery to alleviate stenosis or occlusion, thereby enhancing blood supply via the PCI technique (12–14). Initially, bare metal stents (BMS) marked the inception of stent utilization in clinical practice. However, this advancement was accompanied by the predicament of in-stent restenosis (ISR). The development of drug-eluting stents (DES) has been facilitated with deeper research into the pathology of ISR, improving all aspects of the stent, bringing new alloys that maintain durability and reduce strut thickness to improve deliverability, biocompatible polymers that reduce inflammatory responses and improve drug-eluting kinetics, and a new generation of drugs that predictably inhibit restenosis (15–18). These are designed to reduce endothelial hyperplasia to reduce vascular risk events after stent implantation. Bibliometric analysis serves as a robust investigative method to comprehensively understand the global distribution of countries and regions, prolific authors, research frontiers, and hotspots in the field. By scrutinizing extensive scientific data, including keyword frequencies, and more, this approach unveils intricate development nuances, emergent domains, and interconnected networks, providing clear and intuitive insights for future research trajectories (19, 20). By undertaking a meticulous bibliometric analysis, we seek to unveil the intricate fabric of pertinent countries and regions, prominent research institutions, prolific authors, recurrent keywords, and more, that compose the tapestry of this domain. Furthermore, this analysis endeavors to illuminate research hotspots and cutting-edge domains, aiming to foster innovative perspectives for the future trajectory of DES therapy for CAD.

## Methods and materials

### Data acquisition

The pertinent literature for this study was acquired from the Web of Science Core Collection (WOSCC) through comprehensive searches and downloads, culminating on July 11, 2023. The search query encompassed multiple permutations, including terms like “Drug-Eluting Stents,” “Drug Eluting Stents,” “Stents, Drug-Eluting,” and more, juxtaposed with terms such as “Coronary Artery Disease,” “Artery Disease, Coronary,” and associated variants. This formula was meticulously crafted to ensure inclusivity. The specific search query is as follows:

[TS = (Drug-Eluting Stents) OR TS = (Drug Eluting Stents) OR TS = (Stents, Drug-Eluting) OR TS = (Stents, Drug Eluting) OR TS = (Drug-Eluting Stent) OR TS = (Drug Eluting Stent) OR TS = (Stent, Drug-Eluting) OR TS = (Drug-Coated Stents) OR TS = (Drug Coated Stents) OR TS = (Stents, Drug-Coated) OR TS = (Stents, Drug Coated)] AND [TS = (Coronary Artery Disease) OR TS = (Artery Disease, Coronary) OR TS = (Artery Diseases, Coronary) OR TS = (Coronary Artery Diseases) OR TS = (Left Main Coronary Artery Disease) OR TS = (Left Main Disease) OR TS = (Left Main Diseases) OR TS = (Left Main Coronary Disease) OR TS = (Coronary Arteriosclerosis) OR TS = (Arterioscleroses, Coronary) OR TS = (Coronary Arterioscleroses) OR TS = (Atherosclerosis, Coronary) OR TS = (Atheroscleroses, Coronary) OR TS = (Coronary Atheroscleroses) OR TS = (Coronary Atherosclerosis) OR TS = (Arteriosclerosis, Coronary)].

The scope of literature encompassed the period from January 1, 2002, to July 11, 2023, enlisting records that adhered to English language criteria and were exclusively in the form of reviews and dissertations. The dataset, which spanned the specified time frame, encompassed a total of 5,778 records. These records, each encompassing comprehensive information encompassing authors, titles, sources, publication years, abstracts, keywords, DOI numbers, citation frequencies, and references cited within the respective articles, were amassed.

The resulting data were subsequently exported in plain text format, meticulously curated, and cataloged within a “download_.txt” file for further analysis and scrutiny.

Inclusion exclusion criteria:

1 Inclusion criteria
  (1) Inclusion of all review and dissertation literature on DES for CAD that can be searched by WOSCC from 2002–2023;,
2 Exclusion criteria
  (1) Duplicate published literature;
  (2) Literature type of; conference abstracts, editorial material, conference proceedings, letters, book chapters, briefs, online at tables, revisions, retracted publications, newsletters, news, reprints, retracted content, discussions.
  (3) Literature with incorrect data and for which data could not be extracted;
  (4) Literature with incomplete data provided in the original text and which could not be obtained from the author(s);
  (5) Literature that is not in English, has only an abstract, or for which the full text is not available.

**Figure 1:**
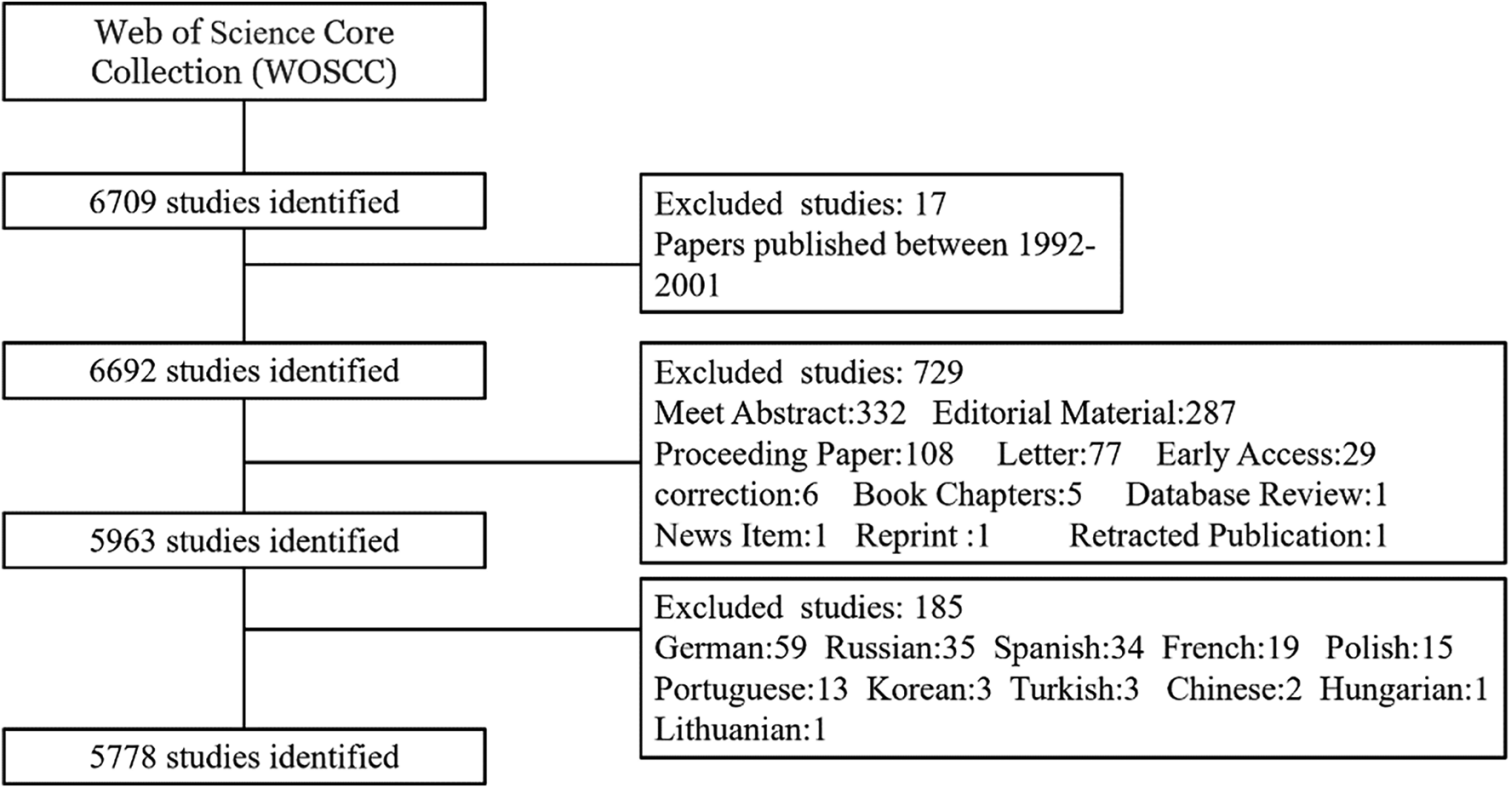
Screening process of WOSCC literature on DES for CAD, 2002–2023.

The screening method is shown in Figure 1.

### Data analysis and visualization

Statistical analysis of the publication data from the literature was conducted employing Excel 2021. This involved calculating key indicators like the yearly article publication count and cumulative publication count to elucidate the research field's trajectory. Furthermore, tables were generated to encapsulate the top 10 countries and top 10 authors in terms of article publication count and citation frequency, thereby facilitating a deeper comprehension of the literature's impact and collaborative dynamics.

To facilitate improved visualization and comprehensive analysis, the advanced edition of CiteSpace 6.2R3 took on responsibilities such as refining, processing, and computing data, along with generating visualization maps (21). The visualization maps, representing cited literature, adopt node size to signify project magnitude, while distinct colors denote various years. The interconnecting lines between nodes mirror collaborative efforts or co-citation associations among projects (22). Employing this approach, the dynamic interplay of node-node connections and the network of citations are observed, illuminating collaboration and citation bonds between projects. Consequently, this methodology facilitates the identification of research hotspots, discerning trends across stages, and projecting potential avenues of future development (23).

VOSviewer 1.6.19 stands as an instrumental tool catering to the visualization and analysis of scientific knowledge (24, 25). Capitalizing on its robust capabilities, this software navigates through country and region, research organization, and keyword co-occurrence analyses (26, 27). By employing this technique, we can shed light on pivotal trends and focal points within the field. This approach renders intricate structures and evolving trends within the research domain comprehensible at a glance, offering an intuitive appreciation of the field's dynamics.

Pajek 5.17, a software renowned for its network analysis and visualization capabilities. Its widespread usage extends to network science and allied disciplines, facilitating an in-depth exploration of complex network structures and attributes (28).

Furthermore, for assessment indicators, citations may take time to accumulate, which makes them not a direct indicator of research activity. For newer publications, the number of citations may not be fully representative of their impact. Focusing on the number of publications allows for a more real-time assessment of research results. So we chose number of publications as the primary indicator for assessment and added citations as a secondary indicator.

**Figure 2:**
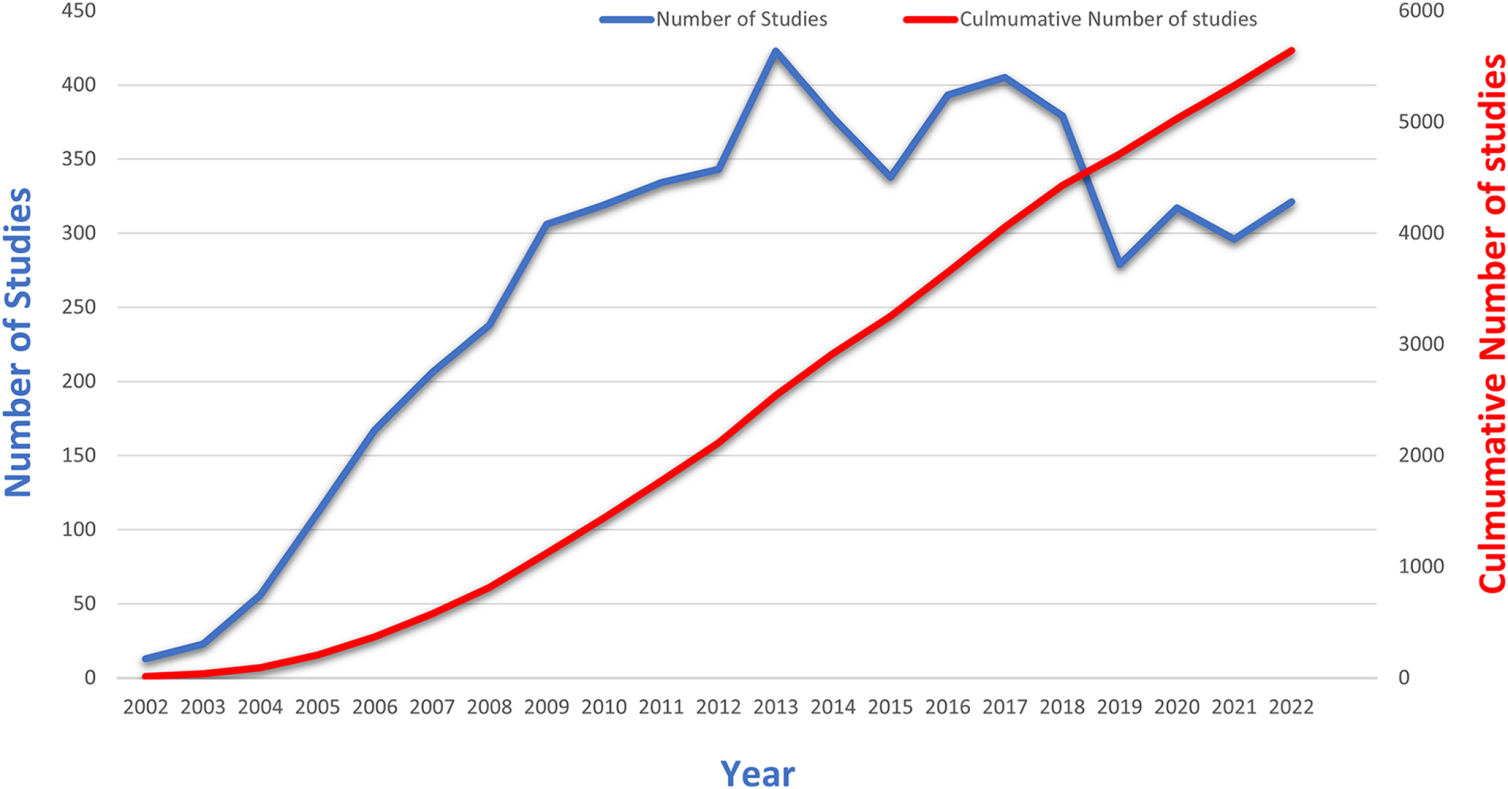
Trends in annual and cumulative number of publications in the WOSCC literature on CAD for DES treatment, 2002–2022.

The analysis and graphing of annual and cumulative publication figures from the WOSCC literature concerning DES therapy for CAD over the past two decades are presented in Figure 2. The graphical representation unmistakably showcases a consistent year-by-year increase in the number of publications from 2002 onward. However, a significant shift in research focus occurred around 2015 due to the introduction of the clinical application of bioresorbable vascular scaffolds (BVS). This pivotal juncture spurred a redirection of attention from DES to BVS.

Nevertheless, the trajectory of BVS was met with nuanced outcomes in light of accumulating clinical evidence. Specifically, the target lesion failure rates—encompassing cardiogenic death, target vessel myocardial infarction, and target lesion revascularization—as well as the risk of in-stent thrombosis demonstrated a noteworthy surge compared to metal DES (29, 30). A comprehensive meta-analysis over a 3-year span underscored that BVS markedly amplified the likelihood of in-stent thrombosis (31).

As a response to these findings, the resurgence of interest in DES was evident post-2016. However, the year 2019 marked the global onset of a novel coronavirus pandemic, significantly reshaping the landscape of medical research. Amid this paradigm shift towards pandemic-related investigations, the number of publications in the domain of DES experienced a temporary downturn. Subsequently, as pandemic-related research stabilized and the epidemic came under control, a gradual rebound in the research output related to DES was witnessed. While the situation regarding the novel coronavirus outbreak has shown signs of improvement, the field has not yet fully recovered to its previous state. This might be attributed to the decrease in stent prices, which, in turn, has resulted in reduced potential revenues for stent companies (32, 33). Along with the rapid growth in the field of structural heart disease interventions, may have diverted the attention of researchers (34, 35).

### National and regional cooperation networks analysis

**Figure 3:**
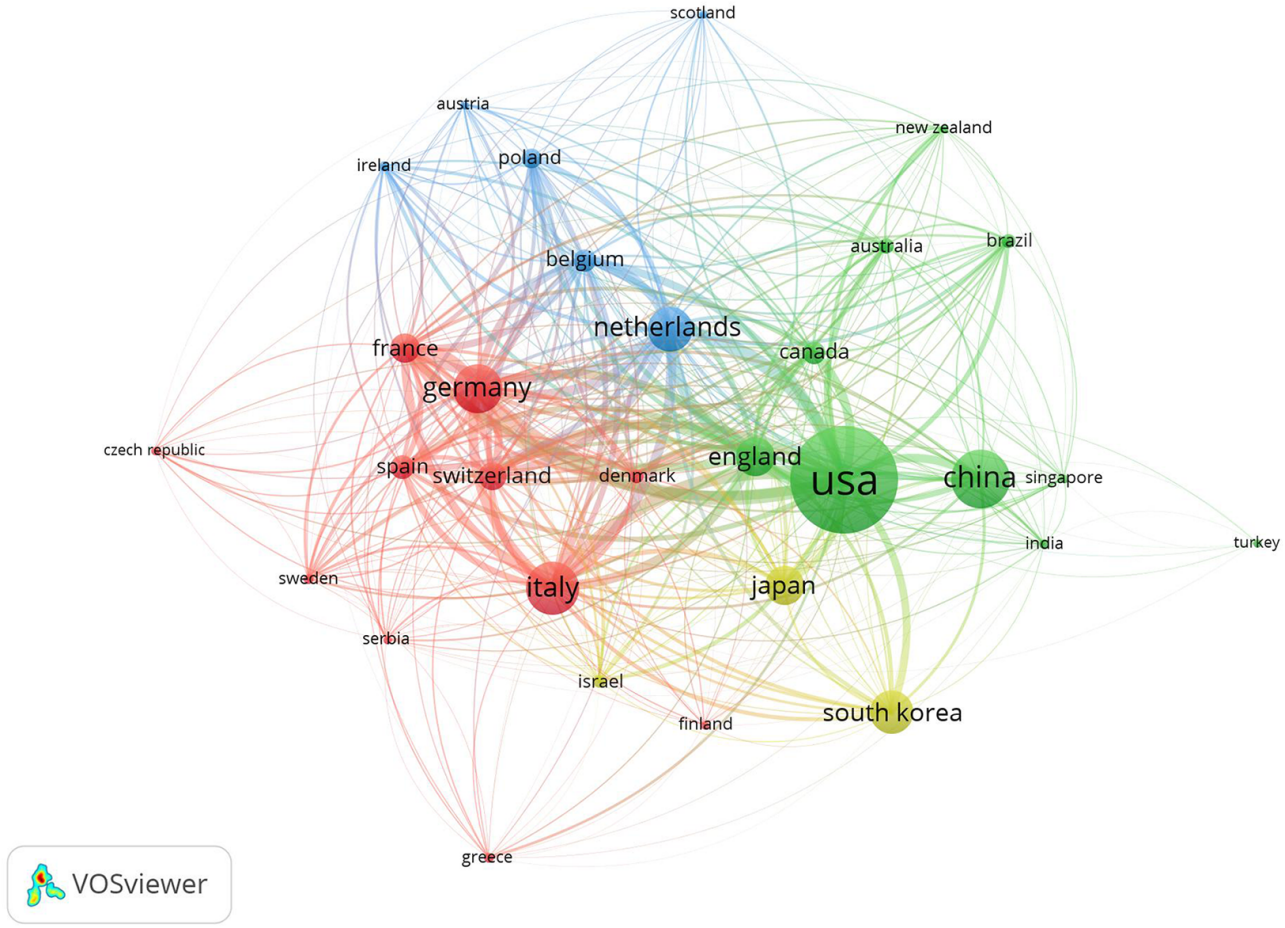
WOSCC national regional collaboration network on DES therapy CAD literature, 2002–2023.

An analysis, utilizing data from WOSCC, was conducted to understand the contributions of different countries to citations. The outcomes are depicted in Figure 3. In this visual representation, the size of each circle corresponds to the number of citations, while the thickness of the lines connecting them signifies the extent of collaboration between the countries.

The top 10 countries, in terms of both publication count and citation impact, have been identified as follows: the United States, Italy, China, Germany, the Netherlands, South Korea, Japan, England, France, and Switzerland. A concise summary of these top countries can be found in Table 1.

**Table 1:** Number of publications and citations from the top 10 countries in the WOSCC literature on CAD for DES, 2002–2023.

Rank	Country	Documents	Citations
1	USA	1,869	105,300
2	Italy	761	35,143
3	China	849	11,284
4	Germany	685	46,993
5	Netherlands	599	46,846
6	South Korea	590	17,222
7	Japan	514	11,265
8	England	511	27,171
9	France	343	29,824
10	Switzerland	332	25,229

Notably, the United States emerges as a dominant force in this field, far surpassing other nations both in terms of publication output and citation impact. The symbiotic relationship between publication count and citation frequency underscores the United States' pivotal role in the field of DES for coronary artery disease, reinforcing its substantial influence on this critical area of research.

### Collaborative network analysis of research institutions

**Figure 4:**
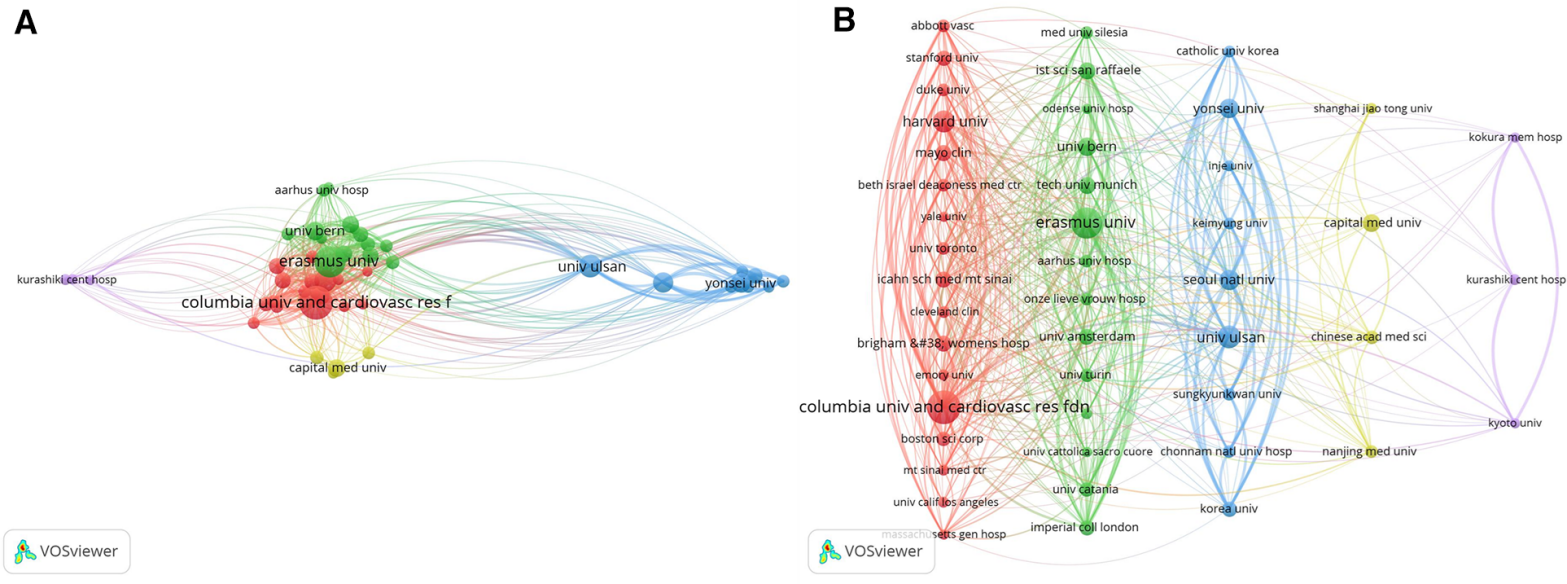
WOSCC collaborative network map of research institutes regarding DES treatment CAD literature, 2002–2023, (**A**) is derived directly from the VOSviewer 1.6.19 and (**B**) is processed by the Pajek 5.17.

Utilizing VOSviewer, a visual mapping of institutional collaborations was meticulously crafted. This intricate map underwent further refinement through the application of Pajek software, culminating in the generation of Figure 4. Five predominant clusters have materialized within this collaboration network, epitomized by Columbia University, Erasmus University Medical Center, University of Ulsan, Capital Medical University, and Kyoto University.

Intriguingly, an observation surfaces: numerous institutions exhibit a lack of connectivity, implying minimal or no cooperative engagement. However, an intriguing possibility surfaces: intermediaries acting as bridges between distinct entities. This proposition seeks to foster collaboration between institutions that may not have direct connections. In essence, leveraging intermediaries to forge connections and bridge gaps between research institutions holds the potential to enhance interdisciplinary communication and foster cooperative endeavors, thereby propelling scientific advancement through synergistic efforts.

Concurrently, the tally of articles published by institutions has been meticulously compiled and is detailed in Table 2. An in-depth analysis of these statistics immediately highlights the prominent position held by Columbia University and the Cardiovascular Research Foundation (CRF) in terms of both article publications and citation frequency. This dominance firmly establishes them as central players in this domain.

**Table 2:** Top 10 WOSCC research institutions in terms of number of publications on DES therapy CAD literature, 2002–2023.

Rank	Organization	Documents	Citations	Country
1	Columbia University & Cardiovascular Research Foundation	354	32,924	USA
2	Erasmus University	310	27,924	Netherlands
3	University of Ulsan	185	7,242	South Korea
4	Harvard Univ	175	22,554	USA
5	Seoul National University	146	3,951	South Korea
6	Yonsei University	141	3,871	South Korea
7	University of Bern	135	15,439	Switzerland
8	Capital Medical University	120	1,356	China
9	Istituto Scientifico San Raffaele	115	9,562	Italy
10	Technical University of Munich	112	8,101	Germany

### Citation network analysis

**Figure 5:**
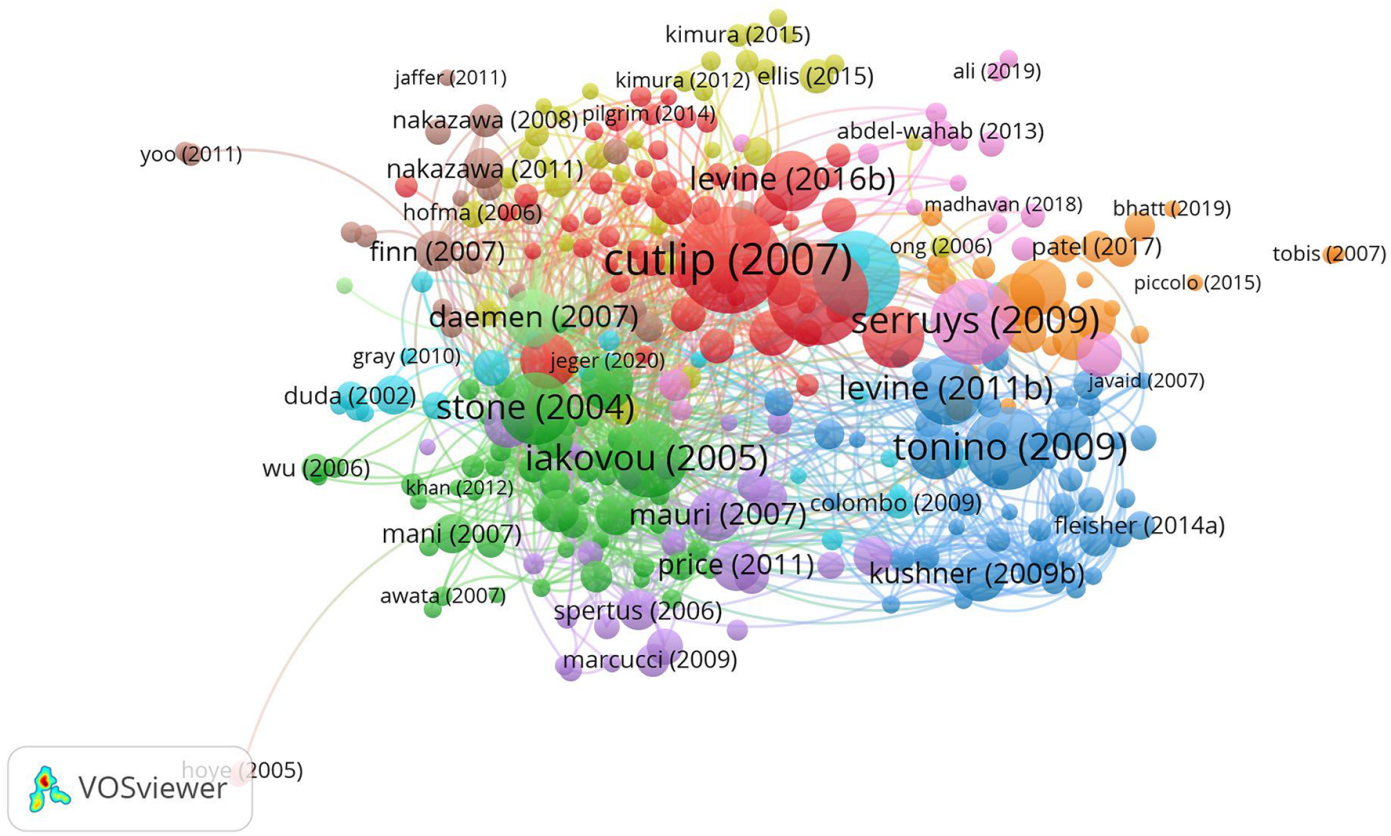
Network diagram of citation relationships in the WOSCC literature on DES treatment of CAD, 2002–2023.

Citation analysis serves as a yardstick for assessing the influence and impact of scholarly works, as illustrated in Figure 5. The top ten cited articles, replete with their titles, affiliated journals, and respective journal impact factors, provide a comprehensive snapshot of the most impactful contributions.

Employing the impact factor (IF) as a metric, the magnitude of a journal's influence is quantified. Larger impact factors denote heightened influence. Furthermore, the Altmetric Score (AS) serves as a metric to gauge the impact of scientific articles in online media (36, 37). While it doesn't gauge the scientific quality of the article, it offers an alternative perspective on the article's impact within the community (38). This insight is explicitly articulated in Table 3. Remarkably, the top ten cited articles predominantly find their home in influential journals such as “Circulation,” “European Heart Journal,” and “New England Journal of Medicine.” This clustering within these high-impact journals underscores their profound sway within the field.

**Table 3:** Top 10 citations, number of citations, journal affiliation, and impact factor of WOSCC literature on DES therapy CAD, 2002–2023.

Rank	Publication	Citations	Journal	IF of Journal	Altmetric score
1	Cutlip et al. (2007) (39)	4,630	Circulation	37.8	31
2	Windecker et al. (2014a) (40)	4,156	European heart journal	39.3	119
3	Neumann et al. (2019a) (41)	3,124	European heart journal	39.3	209
4	Serruys et al. (2009) (42)	2,967	New England journal of medicine	158.5	91
5	Tonino et al. (2009) (43)	2,858	New England journal of medicine	158.5	96
6	Iakovou et al. (2005) (44)	2,461	Jama	120.7	60
7	Stone et al. (2004) (45)	2,147	New England journal of medicine	158.5	12
8	Levine et al. (2011b) (46)	1,991	Journal of the American College of Cardiology	24.0	89
9	Rydén et al. (2013) (47)	1,584	European heart journal	39.3	152
10	Levine et al. (2016b) (48)	1,490	Journal of the American College of Cardiology	24.0	231

A standout article, “Clinical End Points in Coronary Stent Trials: A Case for Standardized Definitions,” emerges as the most cited. Published in “Circulation” in 2007, this seminal work spotlights the imperative for standardized clinical endpoints in coronary stent trials. The article advocates the adoption of two composite endpoints—one device-oriented, encompassing cardiac death, target-vessel myocardial infarction, and target-lesion recanalization; and another patient-oriented, comprising all-cause death, any myocardial infarction, and any recanalization. This harmonization of criteria culminates in consensus, thereby facilitating a robust framework for assessing stent safety and efficacy (39). Nevertheless, the article's AS ranks lower among the top ten articles, likely due to its early online publication when social media wasn't as prevalent.

### Journal double image overlay analysis

**Figure 6:**
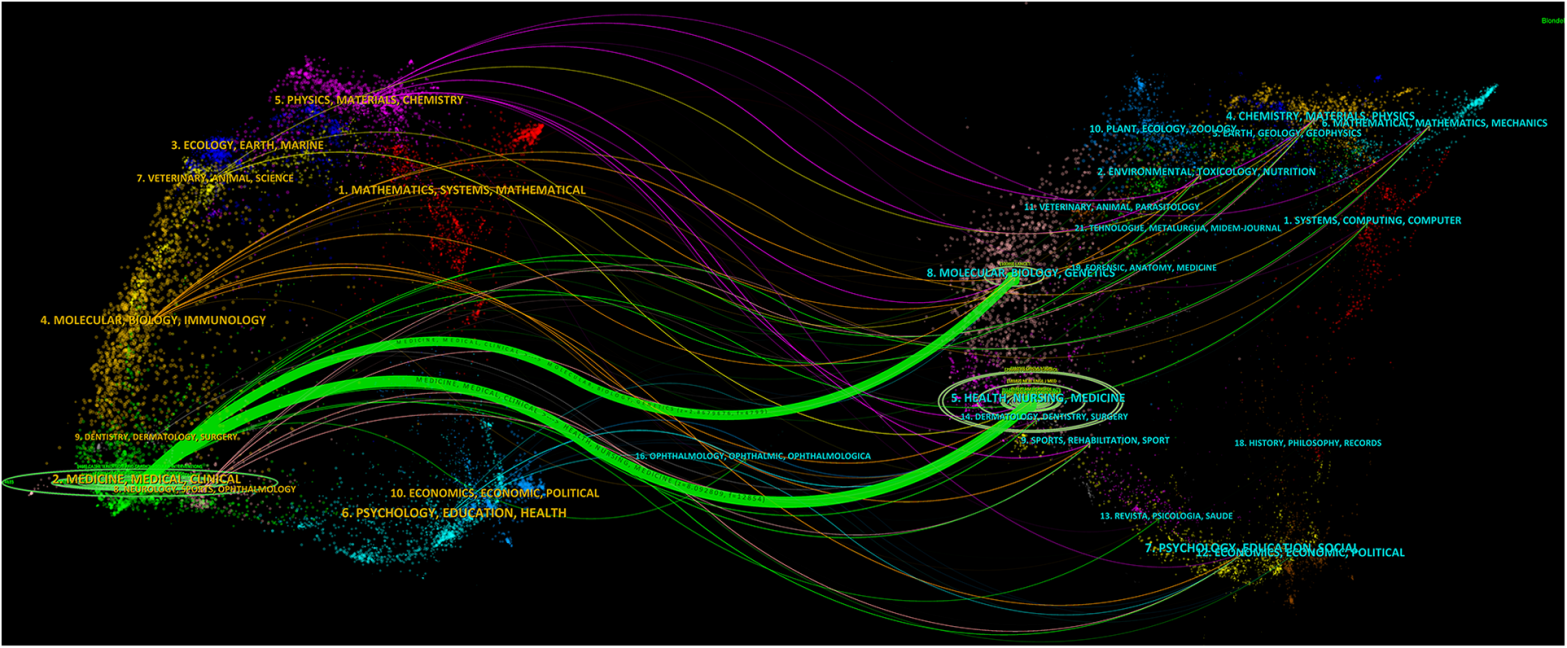
WOSCC double figure overlay of journals relating to DES treatment of CAD, 2002–2023.

The utilization of CiteSpace visualization software for overlay analysis of journal bi-graphs, illustrating citation interrelationships, provides a comprehensive depiction of the intricate citation landscape in the field of DES for coronary artery disease. Figure 6, generated through the “JCR Journal Map” feature and Z-Score function, portrays distinct paths represented by green and orange hues. The left side predominantly encompasses domains like “Medicine,” “Medical,” “Clinical,” “Physics,” “Materials,” and “Chemistry,” while the right side clusters around “molecular biology,” “genetics,” “health,” “nursing,” and “medicine.” Notably, the green links signify significant citation pathways, with an especially robust pathway connecting “Medicine,” “Medical,” and “Clinical” journals to “Health,” “Nursing,” and “Medicine” journals. This pathway, marked by a *Z*-value of 8.092809 and an *F*-value of 12,854, reveals a dense and influential citation network, indicating a dynamic synergy and robust information exchange between disciplines and contributing synergistically to the field's scholarly progression.

### Author network analysis

**Figure 7:**
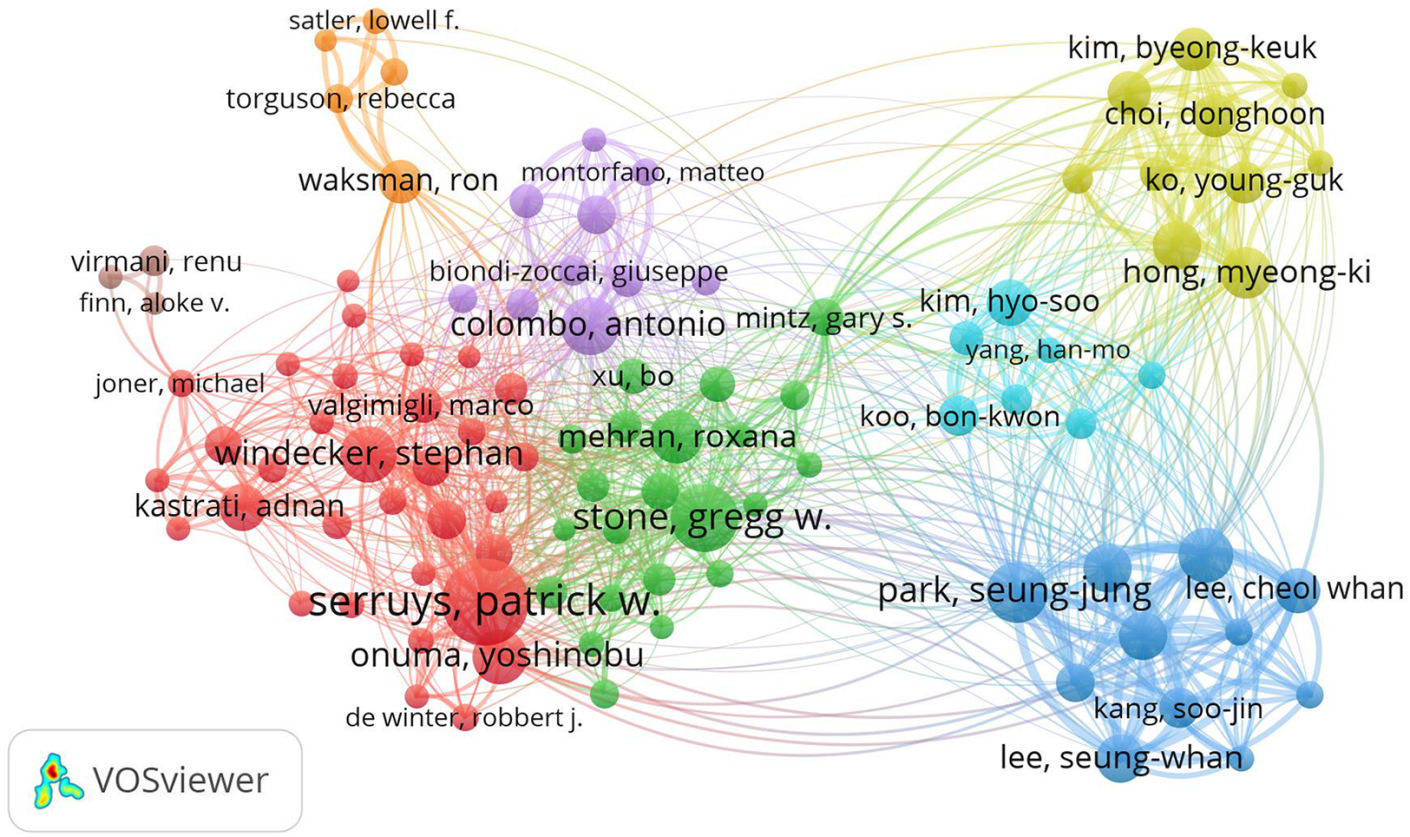
Author relationship network map of literature on DES therapy CAD in WOSCC, 2002–2023.

The outcomes of the comprehensive visualization and analysis of authors within this domain, executed via VOSviewer, are elegantly portrayed in Figure 7. The culmination of this analysis is encapsulated in Table 4, enumerating the top ten authors based on both publication frequency and citation counts. Merging the visual representation with the tabulated data, a clear constellation of leading authors emerges, spotlighting their contributions.The roster of esteemed authors at the helm of publication frequency encompasses luminaries such as Patrick W. Serruys, Gregg W. Stone, Seung-Jung Park, and more.

**Table 4:** Top 10 authors and their number of publications and citations in the field of CAD for DES therapy at WOSCC, 2002–2023.

Rank	Author	Documents	Citations
1	Patrick W. Serruys	233	24,025
2	Gregg W. Stone	158	10,921
3	Seung-Jung Park	136	6,227
4	Antonio Colombo	124	11,348
5	Stephan Windecker	119	13,135
6	Yoshinobu Onuma	113	5,663
7	Duk-Woo Park	112	4,522
8	Roxana Mehran	106	14,233
9	Myeong-Ki Hong	101	3,238
10	Yangsoo Jang	95	2,830

Patrick W. Serruys stands preeminent, boasting a staggering 233 articles to his name, concurrently securing the apex position in terms of citation frequency. Currently holding positions as Professor of Interventional Medicine and Innovation at the National University of Ireland and Cardiology Honorary Professor at the National Heart and Lung Institute, Imperial College London. In addition, he is an honorary professor at the University of Erasmus and most of his works are related to the University of Erasmus. His noteworthy works, such as “A Comparison of Balloon-Expandable-Stent Implantation with Balloon Angioplasty in Patients with Coronary Artery Disease,” “Clinical End Points in Coronary Stent Trials: A Case for Standardized Definitions,” and “Late Thrombosis in Drug-Eluting Coronary Stents after Discontinuation of Antiplatelet Therapy,” showcase his substantial contributions to the field (39, 49, 50).

Patrick W. Serruys' legacy further extends to the pioneering development of DES and an array of interventional procedures and devices, including BVS and transcatheter aortic valve replacement. His profound impact encompasses innovative contributions to vascular imaging techniques like intracoronary near-infrared spectroscopy (NIRS) and optical coherence tomography, propelling the field forward (51–54). His participation in seminal clinical trials like BENESTENT, SYNTAX, and GLOBAL LEADERS has furnished pivotal evidence shaping the evaluation of stent effects and antiplatelet therapy outcomes (55–60).

### Keyword analysis

**Figure 8:**
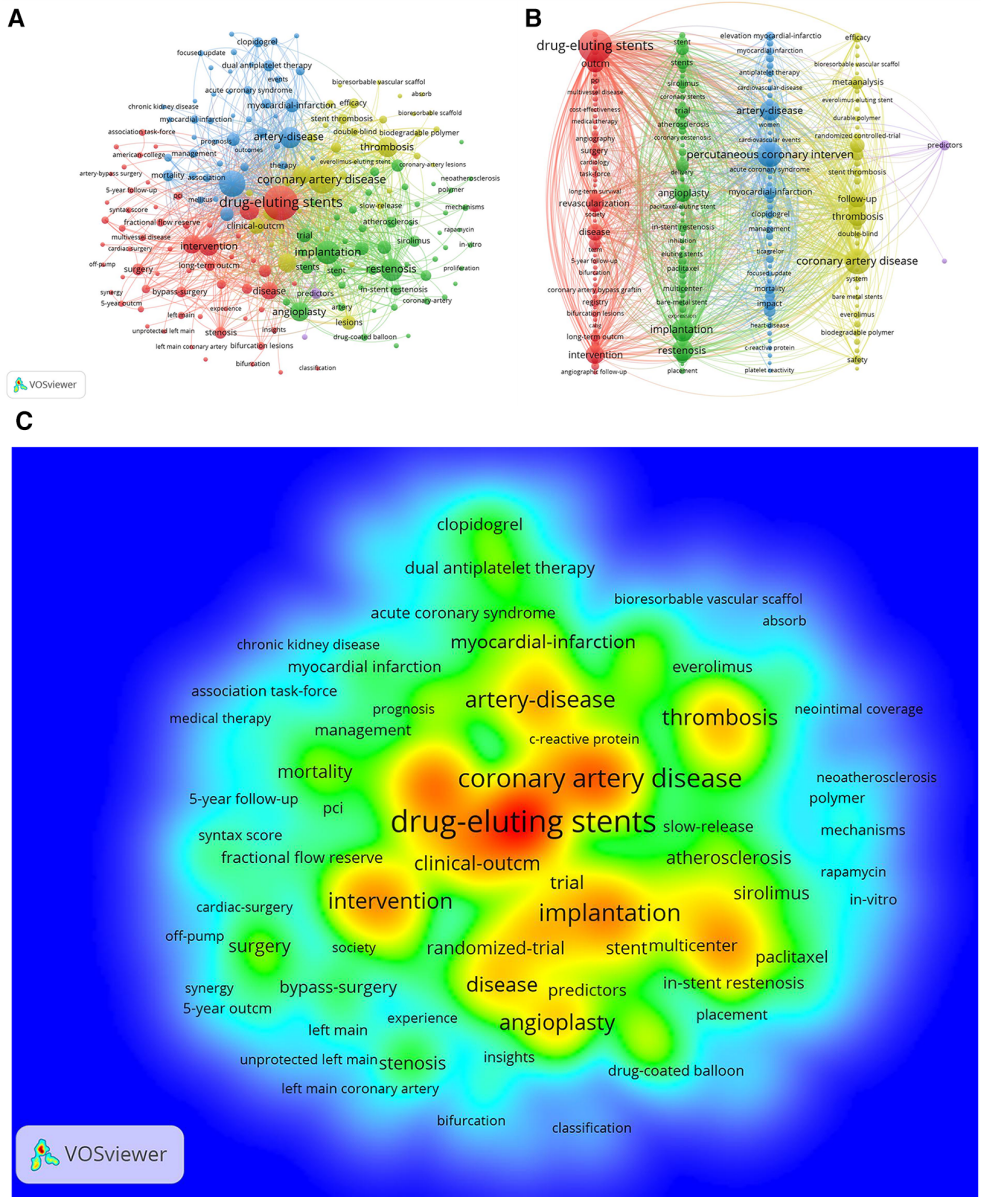
A,B keyword network diagram of the WOSCC literature on DES treatment of CAD, 2002–2023, figure a is derived directly from the VOSviewer 1.6.19 and figure b is processed by the Pajek 5.17. (**C**) Keyword density map of WOSCC literature research on DES for CAD, 2002–2023.

The significance of keyword analysis is vividly depicted in Figures 8A,B illustrating the dynamic interplay and interdisciplinary growth of the field beyond medicine. Utilizing Pajek, Figure 8B visually organizes keywords into clusters, offering a consolidated perspective. The top 10 keywords, including “drug-eluting stents,” “coronary artery disease,” and “percutaneous coronary intervention,” are highlighted in Table 5, emphasizing a focus on understanding coronary restenosis and in-stent thrombosis post PCI. The keyword density chart in Figure 8C reveals that research predominantly centers on the triad of “drug-eluting stents,” “coronary artery disease,” and “clinical outcome,” indicating a concentration on evaluating DES clinical outcomes for CAD treatment and long-term implantation prognosis. Clinical and randomized trials emerge as pivotal methodologies, emphasizing the paramount importance of investigating coronary restenosis and stent thrombosis within this dynamic domain.

**Table 5:** Top 10 keywords appearing in WOSCC literature on DES for CAD, 2002–2023.

Rank	Keyword	Occurrences
1	drug-eluting stents	2,946
2	coronary artery disease	1,785
3	percutaneous coronary intervention	1,677
4	implantation	1,340
5	restenosis	1,208
6	artery-disease	1,106
7	thrombosis	992
8	intervention	925
9	angioplasty	897
10	outcome	870

### Keyword emergence analysis

**Figure 9:**
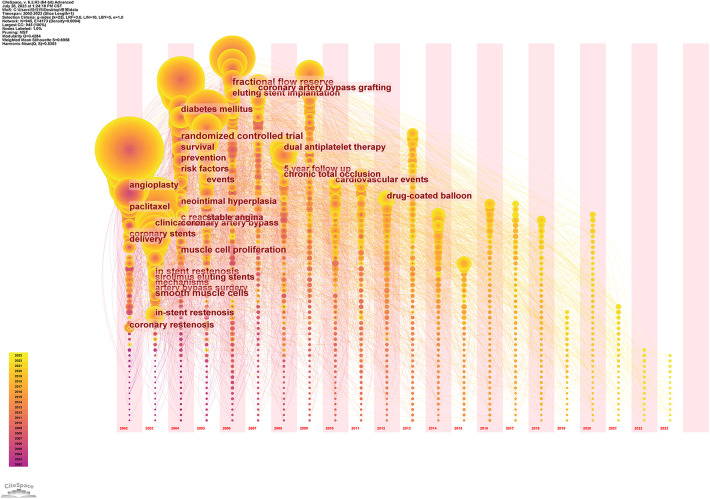
WOSCC keyword time zone map of literature on DES therapy CAD, 2002–2023.

**Figure 10:**
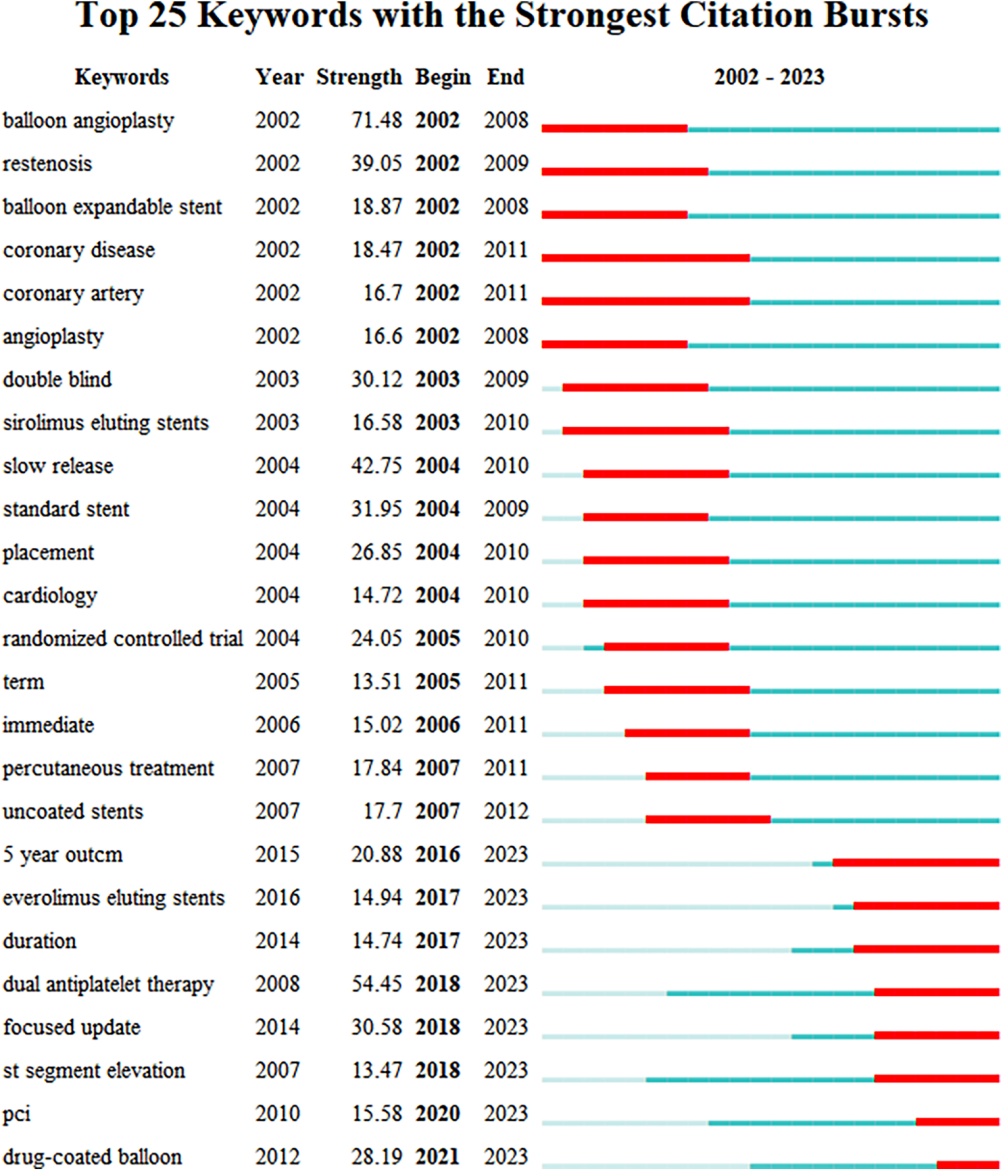
Keyword burst map of WOSCC literature on DES treatment CAD, 2002–2023.

Keyword emergence analysis is a bibliometric-driven methodology designed to unearth keywords that have experienced a noteworthy surge in frequency within a specific timeframe within a given research domain. Leveraging the Citespace system, keyword emergence analysis gauges the intensity and duration of keyword emergence, utilizing diverse metrics. This analysis is then translated into a comprehensive keyword emergence map and corresponding tabulated results, culminating in the identification of the 25 most prominent emergent keywords, as portrayed in Figures 9, 10. This visual and tabular synthesis offers researchers a more intuitive grasp of the trajectory of a particular subject's development and the trajectory of its evolution.

This scrutinized information acts as a compass, deftly directing scholars toward emerging research trajectories and unfolding themes. Delving into Figure 10, it becomes evident that the earliest and most robust keyword is “balloon angioplasty,” while the keywords that continue to surge into 2023 encompass “5-year outcome,” “Everolimus-eluting stents(EES),” “duration,” “dual antiplatelet therapy,” “focus,” and others, such as “antiplatelet therapy,” “focused update,” “segment elevation,” “PCI,” and “drug-coated balloon.”

As shown in Figure 9, the keyword time zone graph can gather the nodes (keywords) in the same time zone at a similar time, which can show how the related research hotspots evolve gradually over time, and this form can clearly show the development of the related research.

## Discussion

The analysis of the annual publication trend within the realm of DES for CAD reveals a upswing in recent years. However, it will take time to determine whether the previous number of postings can be fully restored. Delving into the contribution by countries, it becomes evident that the United States leads the pack in terms of publication volume. When considering institutions, Columbia University and CRF emerges as the frontrunner with the highest count of articles and citations. Notably, the most cited work stems from a paper titled “Clinical end points in coronary stent trials: a case for standardized definitions,” underscoring its influential impact.

Intriguingly, a scrutiny of the dual-map overlay analysis of journals brings forth a robust interaction between those situated in the domains of “Medicine,” “Medical,” and “Clinical,” harmoniously collaborating with counterparts in the spheres of “Health,” “Nursing,” and “Medicine.” This synergy collectively propels the progression of DES for CAD treatment. The examination of prolific authors paints a clear portrait, with Patrick W. Serruys emerging as the predominant figure both in the quantity of publications and the magnitude of citations within the top author cohort.

Examining Figure 10, it is evident that the earliest keyword to emerge was “balloon angioplasty” (angioplasty), marked by the most intense outbreak, which persisted for a span of seven years from 2002 to 2008. In its initial stages, plain old balloon angioplasty (POBA) utilizing balloon catheters demonstrated short-term success. However, long-term prognoses fell short of expectations due to a significant incidence of restenosis. This predicament catalyzed the development of novel techniques and mechanical aids, colloquially referred to as stents, designed to sustain vessel patency post POBA (16). The first-generation solution came in the form of BMS, which served as pioneering interventional therapy stents. BMS managed to mitigate the incidence of restenosis, yet a persistent 20% of cases exhibited restenosis after six months, necessitating recurrent procedures. To address these limitations, DES were conceptualized, leveraging localized drug release to suppress neointimal hyperplasia (16, 61, 62). The advent of DES led to a substantial reduction in restenosis frequency, surpassing the safety and efficacy of radiation therapy or systemic drug administration.

The earliest DES iterations were constructed using stainless steel, featuring a metal substrate, a polymer for controlled antiproliferative drug release, and the antiproliferative drug itself. Subsequent generations ushered in enhancements: thinner struts and refined drug coatings in the second generation, transitioning from sirolimus to everolimus. The third generation introduced ultrathin struts and advanced biodegradable polymer coating technology (63–65). Each progressive DES generation has witnessed an unceasing evolution of technology and design, all with the collective aspiration of achieving heightened long-term safety and efficacy (66).

The keywords that have shown sustained prominence in 2023 include: “5 year outcome,” “Everolimus-eluting stents,” “duration,” “dual antiplatelet therapy,” “focused update,” and more. To enhance analysis, we have categorized similar keywords into the following themes.

### Drug-eluting stents in ST segment elevation myocardial infarction (STEMI)

DES have played a significant role in the management of ST segment elevation myocardial infarction (STEMI), a severe cardiovascular condition often caused by the abrupt blockage of coronary arteries, leading to inadequate blood supply to the heart muscle and subsequent ischemic necrosis. PCI has emerged as the preferred treatment approach for STEMI, offering rapid vessel dilation, restoration of blood flow, reduction of myocardial injury, and potential reduction in mortality and recurrent ischemia rates (41, 67, 68). This technique replaced the conventional pharmacologic therapy and introduced stent-based interventions to the treatment landscape (69–72). The past two decades have witnessed continuous evolution within the realm of DES. The transition from paclitaxel to rapamycin derivatives (such as zotarolimus and everolimus) exemplifies this progression. Furthermore, there have been iterative updates in stent platforms, transitioning from stainless steel to cobalt-chromium (CoCr) and platinum-chromium (PtCr) alloys. Concurrently, novel technologies, ranging from angiography to endovascular imaging, have emerged (63, 73–77). Drug-eluting stent coating technologies have also evolved from permanent polymer coatings to bioabsorbable coatings to polymer-free coatings (63, 78, 79). These advancements have been complemented by insights from pertinent clinical investigations.

The transition from POBA to the use of BMS marked a significant advancement in the field. The introduction of the first-generation DES further revolutionized the approach to treatment. While the initial DES proved effective in reducing adverse events and short-term repeat revascularization compared to BMS, late adverse events such as in-stent thrombosis, reinfarction, and target vessel revascularization (TVR) persisted (80–83).

However, the evolution to second- and third-generation DES has yielded improved outcomes. These advanced DES technologies have led to reductions in the occurrence of adverse events such as TVR and ISR, ultimately enhancing the overall prognosis of patients with STEMI (84–88). The continuous refinement and development of DES have contributed to enhancing patient outcomes and have solidified their pivotal role in the management of ST segment elevation myocardial infarction (89). The assessment of prognostic outcomes between percutaneous coronary intervention with drug-eluting stents (PCI-DES) and coronary artery bypass graft surgery, traditional options for CAD management, reveals comparable long-term results, with no significant differences observed over at least a five-year period (90–92). This finding underscores the importance of offering patients treatment options that yield similar long-term outcomes, allowing for more personalized decision-making in the management of CAD (93).

The evolving landscape of stent technology and PCI procedures holds promise for advancing individualized treatment strategies in coronary artery disease, with tailored surgical protocols and stent selection aiming to optimize outcomes and shape the future of CAD treatment paradigms.

### Dual antiplatelet therapy of drug-eluting stents

Dual antiplatelet therapy (DAPT) plays a crucial role in reducing the risk of thrombosis following DES placement in patients undergoing PCI. This therapeutic approach involves the simultaneous use of two antiplatelet agents, typically aspirin and a P2Y12 receptor blocker like clopidogrel or ticagrelor (94). The duration of this antiplatelet therapy, referred to as “Duration,” is a critical factor that requires careful consideration.

In the context of PCI, DAPT is indispensable for preventing atherothrombotic complications, ensuring the long-term success of the procedure (94–97). However, determining the optimal duration of DAPT for patients with PCI-DES remains a complex challenge (98). Insufficient duration of antiplatelet therapy can heighten the risk of thrombus reocclusion, while excessively prolonged therapy can increase the likelihood of bleeding complications. Therefore, identifying the ideal duration of DAPT is pivotal for achieving favorable outcomes in patients with DES (99–101).

To aid in decision-making regarding DAPT duration post-PCI, the PRECISE-DAPT score was introduced as a five-point scoring system designed to assess the risk of bleeding associated with dual antiplatelet regimens (102). This scoring model assists clinicians in determining the appropriate duration of DAPT following PCI. For patients with acute coronary syndromes who have undergone PCI-DES, a 12-month DAPT regimen is commonly recommended as the standard of care. In contrast, for individuals with chronic coronary artery disease, a treatment approach involving 6 months of DAPT followed by a transition to single antiplatelet therapy (SAPT), typically aspirin, is often advised (97, 103, 104). This tailored approach takes into account the specific clinical characteristics of each patient population, ensuring optimal balance between efficacy and safety in antiplatelet therapy management.

Tailoring antiplatelet therapy adjustment strategies for patients based on their individual risks and clinical characteristics is a critical aspect of managing patients with DES. Various factors such as clinical features, risk factors, type of surgery, and platelet function tests can guide treatment choices and adjustments to strike a balance between the risk of thrombosis or ischemia and the risk of bleeding (105, 106). The two primary strategies for antiplatelet therapy adjustment are “step-down,” aimed at mitigating bleeding risks by transitioning to a less potent agent, and “step-up,” designed to address thrombotic concerns by intensifying therapy through switching to a more potent agent, increasing the dose, or adding a second antiplatelet agent (75, 105, 107–111).

Clinicians face the crucial task of balancing potential benefits and risks in selecting therapeutic strategies for DES treatment, with ongoing research needed to refine late treatment approaches, including the optimal timing and transition between dual and single antiplatelet therapy, to enhance patient outcomes and safety.

### Long-term outcome of drug-eluting stents

The emphasis on the keyword “5 year outcome” indicates that a significant focus of research in the field of DES for CAD is centered around assessing the long-term efficacy and prognosis of these stents. The evaluation of sustained benefits and prevention of late adverse events over a period of at least 5 years is a critical aspect of improving coronary stenting, particularly for first-generation DES implantations (112).

The surge in interest in “Everolimus-eluting stents” highlights a focus on this second-generation drug-eluting stent, utilizing everolimus, categorized as an immunosuppressive drug and belonging to the class of mTOR (mammalian target protein of rapamycin) inhibitors, to counteract vascular endothelial cell proliferation and reduce the risk of ISR (113–115). Everolimus-eluting stents offer diverse designs and materials, facilitating comparisons with other stent types in long-term prognosis studies (85, 116–118). The popularity and versatility of EES in research and clinical practice contribute to their prominence and comprehensive understanding in the field of DES for CAD treatment.

The burst of keywords related to long-term outcomes of DES underscores the significance of understanding the lasting effects of therapeutic interventions. This burst reflects the medical community's recognition of the importance of assessing not only short-term benefits but also the durability of treatment effects over an extended period.

### Hotspots and frontiers

The predicted future research directions and hotspots in the field of DES for CAD are indeed aligned with the evolving needs and advancements in medical research and practice. Ongoing technological innovations introduced into clinical practice hold the promise of enhancing the performance and therapeutic effectiveness of coronary stents, while also providing new insights into the long-term outcomes of coronary interventions.

Optimal update of stent medications: With the integration of new technologies into stent platform development, including nanotechnology and 3D printing, significant advancements are on the horizon. Nanotechnology, for instance, offers the potential to enhance stent coatings and drug delivery systems, ultimately improving stent surface biocompatibility and drug release control. This advancement aims to reduce toxicity and enhance treatment efficacy, with the ultimate goal of decreasing the occurrence of ISR and late-stage vascular risk events (119, 120). On the other hand, 3D printing technology provides the opportunity for individualized treatment options, crafting stents that match the patient's specific vascular characteristics (121).

Optimal update of stent medications: Stent implantation often causes damage to the vascular endothelium. When the process of vascular reendothelialization is not smooth, it can lead to in-stent thrombus formation and restenosis (122, 123). The primary objective of optimizing stent medications is to prevent vascular events by safeguarding endothelial cells and mitigating the adverse impacts on vascular smooth muscle cells and inflammatory cells. This can be achieved through two general approaches: renewing drug combinations and developing novel targeted drugs. A combination of antiproliferative, anti-inflammatory, antithrombotic, and immunosuppressive agents can lead to improved therapeutic outcomes (124). As an illustration, a combination of sirolimus and an antioxidant, which is currently under development, has shown promising results in promoting rapid vascular endothelial cell recovery in an animal model (125). When it comes to the development of novel targeted drugs, this is driven by the fact that current mTOR drugs, like sirolimus and everolimus, lack differential effects on smooth muscle cells and endothelial cells (126, 127). Conversely, microRNA-based cell selection therapy specifically targets vascular smooth muscle cells and inflammatory cells, while safeguarding endothelial cells, leading to a decreased risk of restenosis (127). The development of such targeted drugs represents a future trend.

Additionally, there's a vision of a potential future where smart stents can monitor restenosis and adapt treatment. For instance, these smart stents could measure blood flow using a small ultrasound transducer and adjust the rate of drug release based on the patient's needs to prevent restenosis (128).

In summary, these forthcoming innovations hold the promise of enhancing the safety and effectiveness of coronary interventions.

## Conclusion

Publication volume and citation impact among academic institution. The interdisciplinary nature of DES research is evident through its interaction with journals spanning “Medicine”, “Medical”, and “Clinical” domains, reflecting the collaborative effort to advance healthcare outcomes.

The most cited publication, “Clinical end points in coronary stent trials: a case for standardized definitions,” underscores the importance of standardized criteria in advancing research and clinical practice. Patrick W. Serruys emerges as a prominent author in this field, contributing significantly to its advancement.

Keywords analysis highlights the core themes that underpin the DES research landscape, focusing on critical areas such as DES themselves, coronary artery disease, percutaneous coronary intervention, stent implantation, and restenosis. Keyword burst analysis emphasizes the substantial progress achieved in ST-segment elevation myocardial infarction and long-term outcomes. However, the iterative evolution of stent technology necessitates continued trial data and follow-up to comprehensively assess short- and long-term effectiveness. Looking ahead, the ongoing optimization and updates of stent platforms and medications may usher in new changes, and the introduction of novel stent types promises safer and more effective treatments for patients.
